# Impact of Pharmaceutical Prophylaxis on Radiation-Induced Liver Disease Following Radioembolization

**DOI:** 10.3390/cancers13091992

**Published:** 2021-04-21

**Authors:** Max Seidensticker, Matthias Philipp Fabritius, Jannik Beller, Ricarda Seidensticker, Andrei Todica, Harun Ilhan, Maciej Pech, Constanze Heinze, Maciej Powerski, Robert Damm, Alexander Weiss, Johannes Rueckel, Jazan Omari, Holger Amthauer, Jens Ricke

**Affiliations:** 1 Department of Radiology, University Hospital, LMU Munich, Marchioninistr. 15, 81377 Munich, Germany; ricarda.seidensticker@med.uni-muenchen.de (R.S.); johannes.rueckel@med.uni-muenchen.de (J.R.); jens.ricke@med.uni-muenchen.de (J.R.); 2 Klinik für Radiologie und Nuklearmedizin, Otto-von-Guericke Universitätsklinikum, 39120 Magdeburg, Germany; JannikBeller@web.de (J.B.); maciej.pech@med.ovgu.de (M.P.); constanze.heinze@med.ovgu.de (C.H.); maciej.powerski@med.ovgu.de (M.P.); robert.damm@med.ovgu.de (R.D.); alexander.weiss@med.ovgu.de (A.W.); jazan.omari@med.ovgu.de (J.O.); 3 Department of Nuclear Medicine, University Hospital, LMU Munich, Marchioninistr. 15, 81377 Munich, Germany; andrei.todica@med.uni-muenchen.de (A.T.); harun.ilhan@med.uni-muenchen.de (H.I.); 4 Department of Nuclear Medicine, Charité-Universitätsmedizin Berlin, Corporate Member of Freie Universität Berlin, Humboldt-Universität zu Berlin, and Berlin Institute of Health, Augustenburger Platz 1, 13353 Berlin, Germany; holger.amthauer@charite.de

**Keywords:** radioembolization, radiation induced liver disease, RILD, prophylaxis

## Abstract

**Simple Summary:**

Radioembolization has failed to prove survival benefit in randomized trials, and, depending on various factors including tumor biology, response rates may vary considerably. Studies showed positive correlations between survival and absorbed tumor dose. Therefore, increasing currently prescribed tumor doses may be favorable for improving patient outcomes. The dominant limiting factor for increasing RE dose prescriptions is the relatively low tolerance of liver parenchyma to radiation with the possible consequence of a radiation-induced liver disease. Advances in RILD prevention may help increasing tolerable radiation doses to improve patient outcomes. Our study aimed to evaluate the impact of post-therapeutic RILD-prophylaxis in a cohort of intensely pretreated liver metastatic breast cancer patients. The results of this study as well as pathophysiological considerations warrant further investigations of RILD prophylaxis to increase dose prescriptions in radioembolization.

**Abstract:**

Background: Radioembolization (RE) with yttrium-90 (^90^Y) resin microspheres yields heterogeneous response rates in with primary or secondary liver cancer. Radiation-induced liver disease (RILD) is a potentially life-threatening complication with higher prevalence in cirrhotics or patients exposed to previous chemotherapies. Advances in RILD prevention may help increasing tolerable radiation doses to improve patient outcomes. This study aimed to evaluate the impact of post-therapeutic RILD-prophylaxis in a cohort of intensely pretreated liver metastatic breast cancer patients; Methods: Ninety-three patients with liver metastases of breast cancer received RE between 2007 and 2016. All Patients received RILD prophylaxis for 8 weeks post-RE. From January 2014, RILD prophylaxis was changed from ursodeoxycholic acid (UDCA) and prednisolone (standard prophylaxis [SP]; *n* = 59) to pentoxifylline (PTX), UDCA and low-dose low molecular weight heparin (LMWH) (modified prophylaxis (MP); *n* = 34). The primary endpoint was toxicity including symptoms of RILD; Results: Dose exposure of normal liver parenchyma was higher in the modified vs. standard prophylaxis group (47.2 Gy (17.8–86.8) vs. 40.2 Gy (12.5–83.5), *p* = 0.017). All grade RILD events (mild: bilirubin ≥ 21 µmol/L (but <30 μmol/L); severe: (bilirubin ≥ 30 µmol/L and ascites)) were observed more frequently in the SP group than in the MP group, albeit without significance (7/59 vs. 1/34; *p* = 0.140). Severe RILD occurred in the SP group only (*n* = 2; *p* > 0.1). ALBI grade increased in 16.7% patients in the MP and in 27.1% patients in the SP group, respectively (group difference not significant); Conclusions: At established dose levels, mild or severe RILD events proved rare in our cohort. RILD prophylaxis with PTX, UDCA and LMWH appears to have an independent positive impact on OS in patients with metastatic breast cancer and may reduce the frequency and severity of RILD. Results of this study as well as pathophysiological considerations warrant further investigations of RILD prophylaxis presumably targeting combinations of anticoagulation (MP) and antiinflammation (SP) to increase dose prescriptions in radioembolization.

## 1. Introduction

Radioembolization with yttrium-90 (^90^Y)-loaded resin microspheres (RE, or selective internal radiation therapy [SIRT]) has emerged as an alternative treatment option for patients with primary and secondary liver cancer. However, RE has failed to prove survival benefit in randomized trials, and, depending on various factors including tumor biology, response rates may vary considerably [1,2,3,4,5]. A post hoc analysis of the SARAH trial as well as a competing prospective phase II cohort from another group demonstrated positive correlations between survival and absorbed tumor dose in HCC [6,7]. Recently published smaller single center studies corroborated these observations in metastatic breast and colorectal cancer [8,9,10]. Therefore, increasing currently prescribed tumor doses may often be favorable for improving patient outcomes [11].

Dominant limiting factor for increasing RE dose prescriptions is the relatively low tolerance of liver parenchyma to radiation, which can lead to a deterioration in liver function and even hepatic failure, typically 2 weeks to 4 months after RE [12]. This syndrome is known as radioembolization- or radiation-induced liver disease (RILD). It is characterized by jaundice, development of, or increase in, ascites, hyperbilirubinemia, and hypoalbuminemia in the absence of tumor progression or biliary obstruction [13]. The pathological correlate of radiation-induced liver damage is veno-occlusive disease (VOD). VOD represents an early reaction after radiation exposure and has first been described in patients undergoing external beam radiation therapy. In brief, radiation induced endothelial cell damage followed by inflammation triggers local thrombosis, leading to microvascular flow insufficiency (“sinusoidal congestion”), release of cytotoxic substances and finally hepatocellular necrosis [14,15,16,17]. Preventive drug regimen directed against RILD could mitigate radiation damage and serve developing dose modifications but are still the subject of ongoing research. The current recommendation for RILD prevention after RE is a combination of UCDA and cortisone based on previous work by Gil-Alzugaray et al. [18,19]. One limitation of that regimen is that a considerable number of patients, especially the elderly and diabetic patients, do not tolerate cortisone treatment and discontinue prematurely due to the development of blood sugar changes. In addition, the pathophysiological mechanism behind RILD with VOD does not clearly support the use of cortisone in the acute phase, but rather in the chronic phase to modulate inflammatory mechanisms. A prospective study demonstrated significant mitigation of early focal radiation induced liver injury (fRILI) by post-therapeutic administration of pentoxifylline (PTX), ursodeoxycholic acid (UDCA) and low-molecular weight heparin (LMWH) in patients who underwent image-guided high-dose-rate interstitial brachytherapy of liver metastases [20]. The underlying rationale behind this drug combination is to target acute sinusoidal congestion after radiation by adding an anticoagulant (LMWH) as well as a rheological agent (PTX) to UCDA. UDCA reduces the concentration of potentially hepatotoxic bile acids and presumably downregulates proinflammatory cytokines such as tumor-necrosis factor α (TNF-α) and interleukin-1 [21,22]. 

This study was limited by employing hepatobiliary MRI as a surrogate for focal liver dysfunction after radiation. However, this surrogate has been verified by histopathological proof [23]. Studies have shown that diffuse metastatic disease and prior exposure to chemotherapy increase the risk of RILD [12,18,24,25,26,27]. In liver metastatic breast cancer (LMBC), the degree of liver involvement and liver dysfunction adversely affects patient survival, and LMBC patients that receive RE have generally undergone multiple previous chemotherapies, which makes them a particular risk group to develop RILD after RE [28,29,30,31,32,33]. To determine the efficacy of post-therapeutic RILD prophylaxis with PTX, UDCA and LMWH (modified prophylaxis; MP), we selected a group of intensely pretreated LMBC patients who had received RE during their treatment course. We compared an early cohort undergoing standard prevention including cortisone treatment (standard prophylaxis; SP), and a later group receiving the modified scheme with PTX, UDCA and LMWH (MP). The aim of the study was to see if the different drug regimens have an impact on post-RE liver toxicity and to see if there is a difference in RILD-occurrence. 

## 2. Materials and Methods

### 2.1. Study Design and Population

This was a retrospective analysis of prospectively collected data of patients with LMBC treated with RE at a single center between June 2007 and August 2016. The study which was conducted according to the Helsinki Declaration of 2013 was approved by the local ethics committee (Ethics commission University of Magdeburg: reg. no. IRB00006099, Office for Human Research Protections, Rockville, MD, USA). Written informed consent was waived due to the retrospective nature. 

The decision for RE was taken by a multidisciplinary team. Selection of patients for RE was based on the presence of unresectable hepatic metastases and lack of acceptable chemotherapeutic options (progression after standard chemotherapeutic protocols, therapy discontinuation due to toxicity, patient refusal or for consolidation treatment). Patients had liver-predominant disease, preserved liver function and an acceptable performance status (Eastern Cooperative Oncology Group (ECOG) performance status ≤ 2). Patients with a miliary liver disease pattern were excluded. 

### 2.2. Radioembolization

RE, including pre-procedural diagnostic work-up, was performed according to a standard algorithm as described previously using ^90^Y resin microspheres (SIR-Spheres^®^, Sirtex Medical, Woburn, MA, USA) [34]. This work-up included ^99m^Tc macroaggregated albumin (^99m^Tc-MAA) planar imaging to estimate the lung shunt fraction and SPECT CT imaging to exclude extrahepatic accumulations. The activity of the ^90^Y resin microspheres was calculated by the body surface area (BSA) method. Depending on tumor distribution, ^90^Y resin microspheres were delivered selectively into the hepatic arteries (using a transfemoral approach) in a single session (unilobar or bilobar) or in two sessions as sequential treatment of each lobe 4–8 weeks apart (sequential bilobar). 

### 2.3. RILD Prophylaxis

Between June 2007 and January 2014 all patients undergoing RE received SP consisting of UDCA (oral 500 mg daily; Ursofalk, Falk Pharma, Freiburg, Germany) and prednisolone (oral 5 mg daily) from the day after RE for 8 weeks [18,35,36]. Following the publication of data on the prophylaxis of RILD, the protocol was modified in January 2014. Subsequently, all patients received post-therapeutic modified prophylaxis (MP) with PTX (oral 400 mg three times daily; Trental, Sanofi Aventis, Paris, France), UDCA (oral 250 mg three times a daily) and low-dose LMWH (subcutaneous injection of 40 mg daily (4000 IU); Clexane, Sanofi Aventis, Paris, France) for 8 weeks [20].

### 2.4. Imaging and Volumetry

Baseline iodine contrast agent (90 mL Imeron 300, Iomeprol, Bracco, Princeton, NJ, USA) computed tomography (CT) of the thorax and abdomen (multislice CT, either 16 (Toshiba Aquilion, Toshiba Medical, Tokyo, Japan) or 64 (Siemens Definition AS, Erlangen, Germany) row detector system) and gadolinium ethoxybenzyl diethylenetriamine pentaacetic acid (Gd-EOB-DTPA; Primovist, Bayer Healthcare, Leverkusen, Germany; 0.025 mmol/kg/bodyweight) enhanced magnetic resonance imaging (MRI) of the liver (1.5 Tesla system, Achieva 1.5 T, Philipps, Best, the Netherlands) were available for all patients. Baseline MRI (hepatobiliary phase T1-weighted imaging, 5 mm slice thickness) was used for measurement of the tumor diameters as well as for volumetry of the liver and tumor, using the image processing software Osirix (Antoine Rosset, 2003–2011). Follow-up imaging consisted of either CT of the abdomen (with or without the thorax) or MRI of the liver every 2–4 months. Patients scheduled for sequential bilobar RE underwent MRI on the day of admission for the second RE session.

### 2.5. Toxicity Analysis

Partition model was used for calculation of parenchyma (healthy) liver dose during RE procedure. Routine follow up after RE included standard clinical and laboratory examinations at discharge and every 6 weeks. The National Cancer Institute’s Common Terminology Criteria for Adverse Events (CTCAE) Version 4.02 were used for clinical toxicity assessments. Mild RILD was defined as a bilirubin level ≥ 21 μmol/L and <30 μmol/L; and/or development of ascites. Severe RILD was defined as a bilirubin increase ≥30 μmol/L with or without ascites. Diagnosis of RILD required exclusion of obstructive jaundice and/or hepatic disease progression.

Patients scheduled for sequential bilobar RE treatment occasionally displayed extensive and large scale reduced Gd-EOB-DTPA uptake on MRI in the treated liver lobe before the second RE session, thereby indicating treatment induced subclinical lobar liver dysfunction. In these patients, treatment of the contralateral lobe was cancelled to avoid a global liver failure upon treatment continuation. Such patients were classified as having RILD despite the lack of clinical symptoms.

### 2.6. Response and Survival Analysis

The primary endpoint was toxicity including RILD occurrence. OS and tumor response to treatment were secondary endpoints. Tumor response was assessed by venous phase imaging and recorded according to the Response Evaluation Criteria in Solid Tumors (RECIST) 1.0. Tumor response analyses were based on the best response recorded during follow-up, with patients demonstrating either complete remission (CR), partial remission (PR), stable disease (SD) or progression (PD). OS was defined as the time from the date of first RE session until the death of the patient. 

### 2.7. Statistical Analysis

Statistical analysis was performed using SPSS (SPSS 23, IBM, Armonk, NY, USA). Descriptive analysis of patient and therapy characteristics was performed. Fisher´s exact test was applied for categorical variables and the Mann–Whitney-*U* test for ordinal or continuous variables to identify between-group differences in patients receiving SP or MP. Toxicity was analyzed by Fisher´s exact test and the Wilcoxon rank-sum test, followed by binary logistic and ordinal regressions analysis to test for independent associations between parameters. 

Survival was estimated according to the Kaplan–Meier method. Univariate Cox regression analysis was performed to identify potential pre- and post-therapeutic factors influencing OS. In case of interactions, either the variable with the lower *p*-value in univariate analysis or, if the *p*-value was similar, the clinically more practicable variable was chosen to create multivariate Cox models. Factors found to have an independent impact on survival in the multivariate model were used as stratifying variables in a Kaplan–Meier analysis of survival after the first RE session. The log rank test was used for survival comparison. 

To minimize a potential timeline bias, each group (SP and MP group) was divided in two equal sub-groups and OS was compared. SP was divided into group 1 (GP1SP; treatment period: 13 June 2007–22 June 2010) and group 2 (GP2SP; 23 June 2010–14 January 2014), MP was divided into group 3 (GP3MP; 15 January 2014–28 April 2015) and group 4 (GP4MP; 29 April 2015–31 August 2016).

*p*-Values below 0.05 were considered to indicate statistical significance.

## 3. Results

### 3.1. Population and Treatment Characteristics

A total of 93 patients with LMBC received RE treatment between June 2007 and August 2016 at our institution; 59 patients received SP and 34 patients received MP. Baseline and treatment characteristics are shown in Table 1. The two groups were balanced in terms of tumor load, liver function and previous therapies, RE procedures and the total administered activity during RE. However, mean parenchymal (healthy) liver dose was significantly higher in the MP group (40.2 Gy (12.5–83.5) vs. 47.2 Gy (17.8–86.8), *p* = 0.017) (Table 1). Of the nine patients with planned sequential therapy who discontinued treatment after the first session, treatment of the contralateral lobe was cancelled in three patients due to confluent lobar liver injury after ipsilateral RE, depicted by decreased Gd-EOB-DTPA uptake in MRI before the second treatment session. Even though none of these three patients displayed clinical symptoms, each was counted as RILD (*n* = 2, 3.3% in the SP group; *n* = 1, 2.9% in the MP group). 

The median time to first follow-up was 7.0 (interquartile range (IQR) 6.5–8.0) weeks (IQR).

### 3.2. Toxicity

Baseline and follow-up laboratory parameters are shown in Table 2 and Supplementary Table S1. Differences between the treatment groups at baseline in bilirubin, prothrombin time and aspartate transaminase (ASAT) were within normal range and therefore considered clinically irrelevant. A significant increase in both groups in mean alkaline phosphatase (AP) and ASAT was recorded at follow-up. Alanine transaminase (ALAT) increased significantly in the SP group only. Albumin, cholinesterase, and thrombocytes showed a significant decrease at follow up in both groups. Albumin-bilirubin (ALBI) score significantly increased in both groups. ALBI grade increased in 13 (27.1%) patients in the SP group and 5 (16.7%) patients in the MP group (not significant).

Follow-up data on toxicity were available for 78 of 93 (83.9%) patients. In total, eight cases of RILD of all grades occurred: seven in the SP group, one in the MP group (*p* = 0.143). The incidence of clinical adverse events after RE is shown in Table 3. There were more cases of mild RILD or treatment discontinuations (based on MR depiction of lobar liver dysfunction after previous ipsilateral lobar RE) in the SP group than in the MP group (5 vs. 1; *p* = 0.397). Severe RILD occurred only in the SP group (*n* = 2, 2.6% of overall patients), however, this difference did not reach statistical significance. Regression analysis showed no significant reduction of RILD in the MP group (*p* = 0.145; OR 0.20; 95% CI 0.02–1.73). Only elevated baseline ASAT was a statistically significant predictor of RILD-occurrence in the multivariate regression analysis (*p* = 0.011; OR 17.12; 95% CI 1.93–151.95; Table S2). In addition to RILD, the most common clinical toxicities of any grade were nausea/vomiting (*n* = 13, 16.7%) and abdominal pain (*n* = 12, 15.4%). Nausea/vomiting occurred significantly more often in the MP group than the SP group (9 vs. 4; *p* = 0.026). The incidence of Grade 3 clinical toxicity other than RILD was 3.8% (*n* = 3). There were no Grade 4/5 clinical toxicities. 

In addition, we performed an analysis of descriptors of emerging portal hypertension such as portal vein or spleen diameter changes during follow up. Whereas portal vein and spleen diameter increased in both treatment groups at follow up after 2–4 months, the difference between the MP and the SP group was not significant (Table S3). 

### 3.3. Response and Survival

Best imaging response during follow-up was available in 83 (89.2%) patients. Disease control (CR, PR or SD according to RECIST) was achieved in 74 patients (89.2%) and nine patients (10.8%) had PD. 

Median OS (95% CI) after RE was 8.0 (6.2–9.8) months. Univariate Cox regression for OS identified presence of pre-therapeutic ascites, time from initial diagnosis (ID) of breast cancer to ID of metastatic liver disease, baseline bilirubin, cholinesterase, albumin, CRP, gamma-glutamyltransferase, glutamate dehydrogenase, AP, ASAT, ALAT and MP as factors with a significant impact on survival (Table 4). In multivariate Cox models (after exclusion of interaction), MP (*p* = 0.033; HR 0.47; 95% CI 0.23–0.94) and pre-therapeutic AP ≥ 2 µmol/s.L (*p* = 0.013; HR 2.01; 95% CI 1.16–3.47) showed a significant impact on OS (Table 4). Corresponding Kaplan–Meier survival curves are shown in Supplementary Figures S1 and S2.

To exclude timeline bias, we introduced 2 cohorts for each prophylaxis group (GP1SP, and GP2SP, SP; and GP3MP and GP4MP, MP). GP3MP showed significantly better survival than GP2SP with a median OS (95% CI) of 17.0 (8.55–25.45) months versus 7.0 (5.94–8.06) months (Table 5). Corresponding Kaplan–Meier survival curves are shown in Supplementary Material Figure S3. There was no difference in OS between the subgroups within either prophylaxis group (GP1SP vs. GP2SP and GP3MP vs. GP4MP, respectively).

## 4. Discussion

The applied tumor dose is decisive for patient outcomes in RE. Previous studies have demonstrated the correlation of survival and absorbed tumor dose [6,7,8,9,10]. In addition, variations in response rates after RE indicate a medical need for optimizing dose prescriptions [11]. Preferably, an increase in dose should come with no change of the current safety profile regarding liver failure through RILD, as recent study outcomes of RE, e.g., in HCC, indicate that liver toxicity remains a significant adverse factor negatively influencing patient outcomes [4,10]. Previous reports have defined risk groups for RILD after RE also in non-cirrhotic patients suffering from metastatic disease [12,37,38].

In our retrospective study we sought to determine whether a combination of pentoxifylline (PTX), ursodeoxycholic acid (UCDA) and low-molecular weight heparin (LMWH) positively affects the development of RILD after RE as compared to standard prophylaxis employing cortisone. We employed two cohorts of liver metastatic breast cancer patients who had received either standard prophylaxis with UCDA and cortisone or the modified scheme. Even though the incidence of clinical RILD events most likely was too low to generate a statistical signal, multivariate analyses identified IP an independent variable for improved survival. Furthermore, several factors lead us to hypothesize modified prophylaxis may have been at least as or slightly more effective than standard prophylaxis in preventing liver damage after RE. First, even though both treatment groups appeared to be homogenous, dose exposure of normal liver parenchyma was higher in the modified versus the standard prophylaxis group. Second, the low incidence of events adversely affected statistical proof, despite a relatively higher number of events in the SP group attributable to RILD (such as all grade RILD or ALBI grade increase). The observed survival benefit of patients on modified prophylaxis must be interpreted with caution. Even though both multivariate cox regression as well as Kaplan–Meier curves suggested a robust result, we are unable to explain this outcome. Survival data may have been confounded by unequal distribution of unidentified factors influencing long-term survival in a challenging entity such as metastatic breast cancer. 

Our current data complement results from a previous study, where we were able to show that post-therapeutic application of PTX, UDCA and LMWH for eight weeks significantly reduced the extent and incidence of focal radiation induced liver injury at six weeks after high-dose-rate interstitial brachytherapy of liver metastases [20]. Given the hypothesis generating results of the study described herein as well as the pathophysiology of RILD (which roughly splits the event into venous-occlusive and inflammatory mechanisms), a combination of anticoagulation (such as in the modified scheme) with antiinflammation (such as in the standard prophylaxis) in our view is attractive for further investigations. Based on the low toxicity profile of the modified scheme, this drug combination could be prescribed preventively after radiation therapy (no matter in which form) for at least eight weeks to patients with increased risk of RILD if there are no contraindications. The additional administration of steroids requires individual assessment of the risk of possible side effects but could be of interest in future studies. Another drug used in RILD is defibrotide, a mixture of single-stranded oligonucleotides with a poorly understood effect mechanism. It is approved for the treatment of severe hepatic VOD in patients after bone marrow transplantation [39]. However, this therapy has not yet been tested in patients with RILD after radiotherapy, especially as prophylaxis.

Adverse events from the MP regimen were limited to an increased incidence of nausea and/or vomiting. These symptoms most likely are attributable to PTX, with nausea and vomiting described among its most frequent adverse effects [40]. An important observation of our study was frequent aggravation of the liver function in patients of both groups. Even though several variables remained within normal limits, significant increases of liver enzymes and alkaline phosphatase (AP) were recorded. In addition, albumin, cholinesterase, and thrombocytes showed a significant decrease at follow up. Portal vein and maximum spleen diameter increased significantly. All these variables indicate that despite of preventive measures in both groups, radioembolization adversely affects liver parenchyma and, in most cases subclinical, liver function. It is uncertain whether these effects in long-term may limit subsequent tumor therapies and therefore survival. 

The study had several limitations, such as those inherent in its retrospective design and small sample size, inconsistency in data collection, and the lack of randomization that could have introduced a timeline bias. No follow-up toxicity data were available for 15 patients. In addition, there were not enough events (mild or severe RILD) to reach statistical significance. Data on the development of portal hypertension by portal vein and spleen diameter measurements were limited to a time frame of up to four months only. Previous observations suggest a possible later onset of portal hypertension in selected patients undergoing RE [41].

Our findings may stimulate further investigations in liver protective medication not only with RE, but also in other high conformal irradiation techniques of the liver. In the last decade, the introduction of RE as well as advanced high conformal precision radiotherapy such as stereotactic body irradiation or CT-guided brachytherapy has boosted scientific and clinical interest in liver irradiation. A key driver has been that these techniques enable better targeting and sparing of healthy liver tissue, thus allowing delivery of substantial tumor doses despite the relatively low tolerance of liver parenchyma to radiation [15,42,43,44]. 

## 5. Conclusions

RE and high conformal liver irradiation techniques are limited by potential adverse RILD development. RILD prophylaxis with PTX, UDCA and LMWH appears to have an independent positive impact on OS in patients with metastatic breast cancer after RE compared to prophylaxis with UDCA and prednisolone and may reduce the frequency and severity of RILD. However, mild or severe RILD events proved rare in the overall cohort, which limits the value of the results. Both drug regimens employed for post-therapeutic RILD prophylaxis were well tolerated. Results of this study as well as pathophysiological considerations warrant further investigations of RILD prophylaxis presumably targeting combinations of anticoagulation (MP) and antiinflammation (SP). In effect, advances in RILD prevention may help increasing tolerable RE or liver irradiation doses to improve patient outcomes.

## Figures and Tables

**Table 1:** Baseline and treatment characteristics.

Characteristic	Standard Prophylaxis (*n* = 59)		Modified Prophylaxis (*n* = 34)		*p*-Value
Female sex	59	(100.0)	34	(100.0)	
Age, years	56.0	(49.0–67.0)	55.0	(51.0–64.0)	0.861
Age ≤ 60 years	36	(61.0)	23	(67.6)	0.656
**Primary tumor**					
Estrogen receptor positive	46	(79.3)	27	(79.4)	1.000
Progesterone receptor positive	40	(69.0)	21	(61.8)	0.501
Hormone receptor positive ^a^	48	(81.4)	28	(82.4)	1.000
Her2 neu positive ^b^	13	(22.4)	3	(8.8)	0.153
TNBC ^c^	9	(15.3)	5	(14.7)	1.000
Grading ^d^					
1	1	(1.7)	1	(2.9)	1.000
2	33	(55.9)	23	(67.6)	0.368
3	22	(37.2)	9	(26.5)	0.357
**Metastatic disease**					
Time ID breast cancer to ID LMBC, years	4.0	(1.0–6.0)	3.0	(1.0–8.0)	0.810
Time ID metastatic liver disease to RE, months	19.0	(9.0–37.0)	15.0	(6.0–29.0)	0.127
Tumor load, %	13.0	(3.9–24.0)	10.0	(4.0–20.0)	0.263
Extrahepatic metastases	42	(71.2)	27	(79.4)	0.465
**Previous therapies**					
Chemotherapy	58	(98.3)	34	(100.0)	1.000
Number of chemotherapy lines	3	(2–4)	4	(2–5)	0.163
Local therapy of liver metastases	19	(32.2)	6	(17.6)	0.151
Surgery	6	(10.2)	1	(2.9)	0.416
Interstitial brachytherapy	9	(15.3)	4	(11.8)	0.762
Radiofrequency ablation	2	(3.4)	1	(2.9)	1.000
TACE	1	(1.7)	0	(0.0)	1.000
**Clinical data**					
ALBI grade					
1	53	(89.8)	31	(91.2)	1.000
2	6	(10.2)	3	(8.8)	1.000
3	0	(0.0)	0	(0.0)	
Total liver volume, ml	1644	(940–3323)	1532	(1060–3659)	0.109
**Radioembolization procedure**					
Bilobar sequential	43	(72.9)	22	(64.7)	0.484
Bilobar	2	(3.3)	1	(2.9)	1.000
Unilobar right	13	(22.0)	10	(29.4)	0.461
Unilobar left	0	(0.0)	0	(0.0)	
Superselective ^e^	1	(1.7)	1	(2.9)	1.000
Mean (range) administered activity first session, MBq	1007	(540–1900)	1080	(490–1990)	0.126
Mean (range) administered activity second session, MBq	666	(250–1250)	730	(400–1290)	0.565
Mean (range) total administered activity, MBq	1493	(800–2700)	1528	(650–1990)	0.317
Mean (range) parenchymal liver dose, Gy	40.2	(12.5–83.5)	47.2	(17.8–86.8)	0.017
**Therapy discontinuation after first session**	6	(10.2)	3	(8.8)	1.000
Massive disease progression	2	(3.3)	1	(2.9)	1.000
Hepatic injury (imaging based)	2	(3.3)	1	(2.9)	1.000
Patient withdrew from study	1	(1.7)	1	(2.9)	1.000
Technical reasons	1	(1.7)	0	(0.0)	1.000
Chemotherapy during follow-up	11	(18.6)	2	(5.9)	0.069
Best response ^f,g^					
Complete remission	1	(1.7)	1	(2.9)	1.000
Partial remission	37	(62.7)	22	(64.7)	0.621
Stable disease	7	(11.9)	6	(17.6)	0.759
Progressive disease	5	(8.5)	4	(11.8)	1.000
Lost to follow-up	6	(10.2)	1	(2.9)	0.416

Values presented are no. (percentage) for categorical and median (interquartile range) for ordinal and continuous variables if not marked otherwise. Proportion analysis tests for categorical variables were performed using the Fisher’s exact test. Nonparametric tests for ordinal and continuous variables were performed using the Mann–Whitney *U* test. *p* < 0.05 values indicate statistical significance. ^a^ Estrogen- and/or progesterone receptor positive; ^b^ at least IHC-Score +3; ^c^ triple-negative breast cancer, estrogen receptor, progesterone receptor and Her2 neu negative; ^d^ missing values: Grading, 4/93; ^e^ embolization of segmental branch of hepatic artery; ^f^ Response Evaluation Criteria in Solid Tumors (RECIST); ^g^ missing values: Best response, 10/93 ID, initial diagnosis; LMBC, liver metastases; breast cancer; RE, radioembolization; TACE, transarterial chemoembolization; ALBI, albumin-bilirubin grade; MBq, megabecquerel; Gy, Gray.

**Table 2:** Laboratory parameters at baseline and follow-up (Part I; Part II see Supplementary Material Table S1).

Variable (Normal Range)		Standard Prophylaxis (*n* = 48)	Modified Prophylaxis (*n* = 30)	*p*-Value (Between Group) ^a^	*p*-Value (Baseline vs. Follow-Up) ^b^
Bilirubin (<21 µmol/L)	Baseline	7.5 ± 2.7	6.5 ± 3.8	0.035	
	Follow-up	13.3 ± 22.3	9.4 ± 11.3	0.195	0.121/0.031
	Δ	5.7 ± 22.1	2.9 ± 9.9	0.510	
ALBI score	Baseline	−3.1 ± 0.3	−3.1 ± 0.3	0.541	
	Follow-up	−2.7 ± 0.6	−2.8 ± 0.5	0.251	<0.001/0.003
	Δ	0.4 ± 0.5	0.2 ± 0.4	0.144	
ALBI grade increase FU (*n*)		13 (27.1%)	5 (16.7%)	0.408	

Mean values ± 1 SD; Δ: (Follow-Up)—(Baseline), Relative Δ: ((Follow-Up)—(Baseline))/(Baseline); ALBI Score, albumin-bilirubin score: (log10 bilirubin (µmol/L) × 0.66) + (albumin (g/L) × −0.0852); ALBI grade: grade 1: ≤−2.60, grade 2: −2.60 to (−1.39), grade 3: >−1.39; FU, follow-up; *p* < 0.05 indicates statistical significance. ^a^ Between group comparison, Mann–Whitney U test; ^b^ Wilcoxon Test Comparison versus baseline, Wilcoxon test (standard prophylaxis/modified prophylaxis).

**Table 3:** Toxicity analysis.

Variable	Standard Prophylaxis (*n* = 48)		Modified Prophylaxis (*n* = 30)		*p*-Value
RILD (of all grades)	7	(14.6)	1	(3.3)	0.143
Mild RILD (bilirubin ≥ 21 µmol/L or ascites)	5	(10.4)	0	(0.0)	0.150
Severe RILD (bilirubin ≥ 30 µmol/L and ascites)	2	(4.3)	0	(0.0)	0.520
Discontinuation of treatment due to liver injury (image based)	0	(0.0)	1	(3.3)	0.358
Follow-up ascites	8	(16.7)	4	(13.3)	0.758
Perihepatic amount of ascites, mm (mean, range)	3	(0–45)	1	(0–20)	0.165
Requiring treatment	3	(6.3)	0	(0.0)	0.281
Complications apart from RILD (CTCAE 1 + 2/3/4)	21 (19/2/0)		19 (19/1/0)		
Abdominal pain	8	(16.7)	4	(13.3)	0.758
Dyspepsia	3	(6.3)	2	(6.7)	1.000
Fever	1	(2.1)	0	(0.0)	1.000
Fatigue	1	(2.1)	2	(6.7)	0.555
Nausea/vomiting	4	(8.3)	9	(30.0)	0.026
Others ^a^	7	(14.6)	6	(20.0)	0.548

Values are no. (percentage) for categorical and median (interquartile range) for ordinal and continuous variables if not marked otherwise. Proportion analysis tests for categorical variables were performed using the Fisher’s exact test. Nonparametric tests for ordinal and continuous variables were performed using the Mann–Whitney *U* test. *p* < 0.05 indicates statistical significance. ^a^ Bitter taste, vessel injury, ulceration, leucocytosis, allergic reaction, hematoma, vertigo, headache, presyncope; RILD, radiation induced liver disease; Common Terminology Criteria for Adverse Events (CTCAE), v4.0.

**Table 4:** Univariate and multivariate Cox regression analysis for overall survival following radioembolization.

Variable	Univariate Analysis		Multivariate Analysis	
	*p*-Value	Hazard Ratio (95% CI)	*p*-Value	Hazard Ratio (95% CI)
Age ≤ 60	0.581	1.15 (0.70–1.91)		
Estrogen receptor positive	0.839	1.06 (0.59–1.93)		
Progesterone receptor positive	0.628	1.14 (0.68–1.92)		
Hormone receptor positive ^a^	0.441	1.29 (0.67–2.49)		
Her2 neu positive ^b^	0.278	1.39 (0.77–2.52)		
TNBC ^c^	0.665	0.86 (0.42–1.74)		
Grading G3 ^d^	0.412	0.82 (0.51–1.31)		
Time ID breast cancer to ID LMBC ≥2 years	0.033	0.58 (0.35–0.96)	0.118	0.66 (0.40–1.11)
Time ID metastatic liver disease to RE ≥1 year	0.265	1.35 (0.80–2.28)		
Tumor load ≥ 5%	0.636	1.15 (0.65–2.03)		
Maximum diameter of liver metastases ≥3.9 cm	0.109	1.51 (0.91–2.49)		
Pretherapeutic extrahepatic disease (EHD)				
Lung is one of the metastatic sites	0.657	1.18 (0.58–2.40)		
Bone metastases only	0.386	1.25 (0.76–2.06)		
EHD without bone metastases	0.718	0.86 (0.39–1.91)		
Bone metastases plus at least one other metastatic site	0.140	1.62 (0.85–3.08)		
Pretherapeutic ascites	0.005	3.89 (1.52–9.93)	0.118	2.16 (0.82–5.69)
Pretherapeutic chemotherapy ≥ 3 lines	0.582	0.87 (0.53–1.42)		
Presence of hepatic steatosis	0.361	1.33 (0.72–2.46)		
High-grad hepatic steatosis	0.460	1.32 (0.64–2.73)		
Prior local therapies of liver metastases	0.748	0.91 (0.53–1.58)		
Surgery	0.207	0.51 (0.18–1.45)		
Interstitial brachytherapy	0.600	1.20 (0.61–2.39)		
Radiofrequency ablation	0.598	0.73 (0.23–2.35)		
Bilirubin ≥ 21 µmol/L	0.011	15.34 (1.85–127.38)	^f^	
Cholinesterase ≤ 88 µmol/s.L	0.056	2.30 (0.98–5.42)	^e^	
Albumin ≤ 35 g/L	0.001	18.20 (3.53–93.81)	^e^	
C-reactive protein ≥ 5 mg/dL	<0.001	2.70 (1.59–4.60)	^e^	
Gamma glutamyltransferase ≥1.19 µmol/s.L	<0.001	4.49 (2.48–8.14)	^e^	
Glutamate dehydrogenase ≥120 µmol/s.L	0.015	1.85 (1.13–3.05)	^e^	
Alkaline phosphatase ≥ 2 µmol/s.L	<0.001	2.68 (1.59–4.52)	0.013	2.01 (1.16–3.47)
Aspartate transaminase ≥ 0.83 µmol/s.L	<0.001	3.25 (1.92–5.52)	^e^	
Alanine transaminase ≥ 0.83 µmol/s.L	0.007	2.04 (1.21–3.42)	^e^	
Thrombocytes ≤ 146 Gpt/L	0.933	0.95 (0.30–3.05)		
ALBI Grade > 1	0.130	1.80 (0.84–3.85)		
Bilobar RE	0.330	0.76 (0.44–1.32)		
Unilobar RE	0.215	1.42 (0.82–2.49)		
Total administered activity ≥ 1500 MBq	0.339	0.79 (0.48–1.29)		
Normal liver dose ≥ 40 Gy	0.013	0.53 (0.33–0.88)		
Chemotherapy during follow-up	0.154	1.63 (0.83–3.18)		
Modified prophylaxis	0.004	0.37 (0.19–0.73)	0.033	0.47 (0.23–0.94)

Cox regression analysis was performed. *p* < 0.05 indicates statistical significance. ^a^ estrogen- and/or progesterone receptor positive; ^b^ at least IHC-Score +3; ^c^ triple-negative breast cancer: estrogen receptor, progesterone receptor and Her2 neu negative; ^d^ missing value—Grading. 4/78; ^e^ not included in the multivariate model due to interactions with alkaline phosphatase; ^f^ not included in the multivariate model due to interactions with pretherapeutic ascites; TNBC, triple-negative breast cancer; ID, initial diagnosis; LMBC, liver metastases breast cancer; EHD, extrahepatic disease; ALBI, albumin-bilirubin grade; RE, radioembolization; MBq, megabecquerel.

**Table 5:** Overall survival depending on time-point of treatment.

Prophylaxis	Treatment Period	Group Assignment	Median Overall Survival after RE ^a^	GP1SP vs. GP2SP ^b^	GP2SP vs. GP3MP ^b^	GP3MP vs. GP4MP ^b^
Standard prophylaxis (*n* = 59)	13 June 2007–22 June 2010	Group 1 (GP1SP, *n* = 29)	8.00 (6.96–9.04)			
	23 June 2010–14 January 2014	Group 2 (GP2SP, *n* = 30)	7.00 (5.94–8.06)	*p* = 0.546		
Modified prophylaxis (*n* = 34)	15 January 2014–28 April 2015	Group 3 (GP3MP, *n* = 17)	17.00 (8.55–25.45)		*p* = 0.001	
	29 April 2015–31 August 2016	Group 4 (GP4MP, *n* = 17)	Not reached			*p* = 0.252

To exclude time-line bias 2 cohorts per each prophylaxis group were formed with reference to the time point of treatment. Between-group overall survival was compared (group1 (GP1SP) and group 2 (GP2SP) received standard prophylaxis, group 3 (GP3MP) and group 4 (GP4MP) received modified prophylaxis). Overall survival comparing group 2 to group 3 is significantly different only indicating that the group effect is stronger than possible training effects. *p* values indicate statistical significance. ^a^ Kaplan–Meier analysis, median (95%CI); ^b^ Log rank test was used for comparison of group survival. RE, radioembolization. GP, group.

## Data Availability

Anonymized study data are available from the corresponding author upon reasonable request.

## Supplementary Materials

The following are available online at https://www.mdpi.com/article/10.3390/cancers13091992/s1, Figure S1: Overall survival separated by prophylaxis group, Figure S2: Overall survival separated by baseline alkaline phosphatase levels, Figure S3: Overall survival depending on time-point of treatment, Table S1: Laboratory parameters at baseline and follow-up (Part II), Table S2: Predictors of RILD^1^ (*n* = 78), Table S3: Changes of portal vein and maximal spleen diameter as hallmarks of portal hypertension post RE.
