# Supplementary material for: Impact of Pharmaceutical Prophylaxis on Radiation-Induced Liver Disease Following Radioembolization
Source: Cancers (Basel). 2021 Apr 21;13(9):1992. doi: 10.3390/cancers13091992 (PMC8122451; doi:10.3390/cancers13091992)
Supplement: Supplementary file 1 [file cancers-13-01992-s001.zip › cancers-1195370-supplementary.pdf]

cancers

MDPI

Supplementary Materials: Impact of Pharmaceutical Prophylaxis on Radiation-Induced Liver Disease Following Radioembolization

Max Seidensticker, Matthias Philipp Fabritius, Jannik Beller, Ricarda Seidensticker, Andrei Todica, Harun Ilhan, Maciej Pech, Constanze Heinze, Maciej Powerski, Robert Damm, Alexander Weiss, Johannes Rueckel, Jazan Omari, Holger Amthauer and Jens Ricke

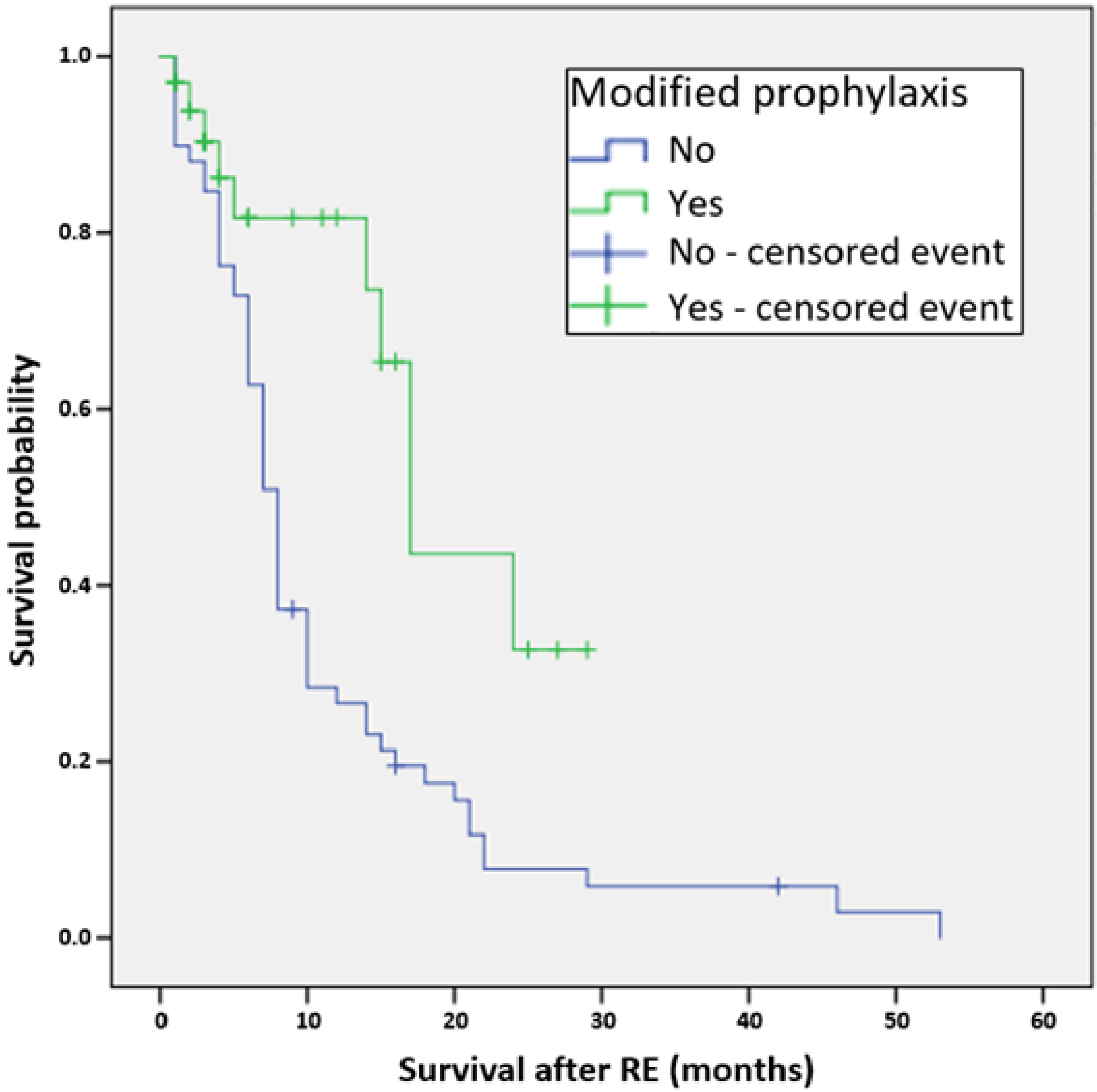

Figure S1. Overall survival separated by prophylaxis group. Kaplan Meier survival analysis calculated from the date of radioembolization. Median survival (95% CI) was 17.0 (14.3–19.7) months in the modified prophylaxis group versus 8.0 (7.0–9.0) months in the standard prophylaxis group. RE, radioembolization.

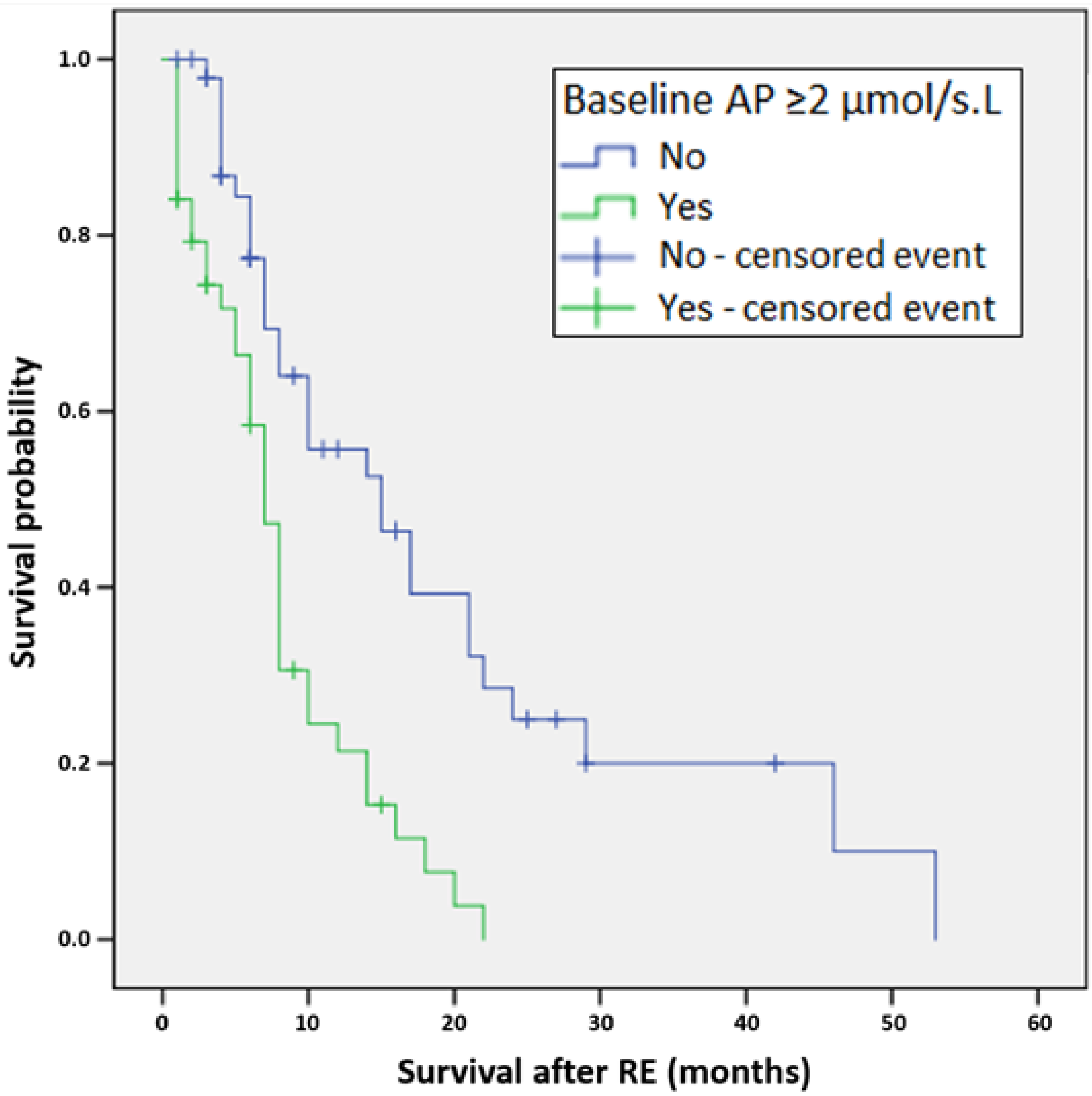

Figure S2. Overall survival separated by baseline alkaline phosphatase levels. Kaplan Meier survival analysis calculated from the date of radioembolization. Median survival (95%CI) with baseline AP ≥ 2 µmol/s.L was 7.0 (5.7–8.1) versus 15.0 (8.1–21.9) with baseline AP < 2 µmol/s.L. RE, radioembolization; AP, alkaline phosphatase.

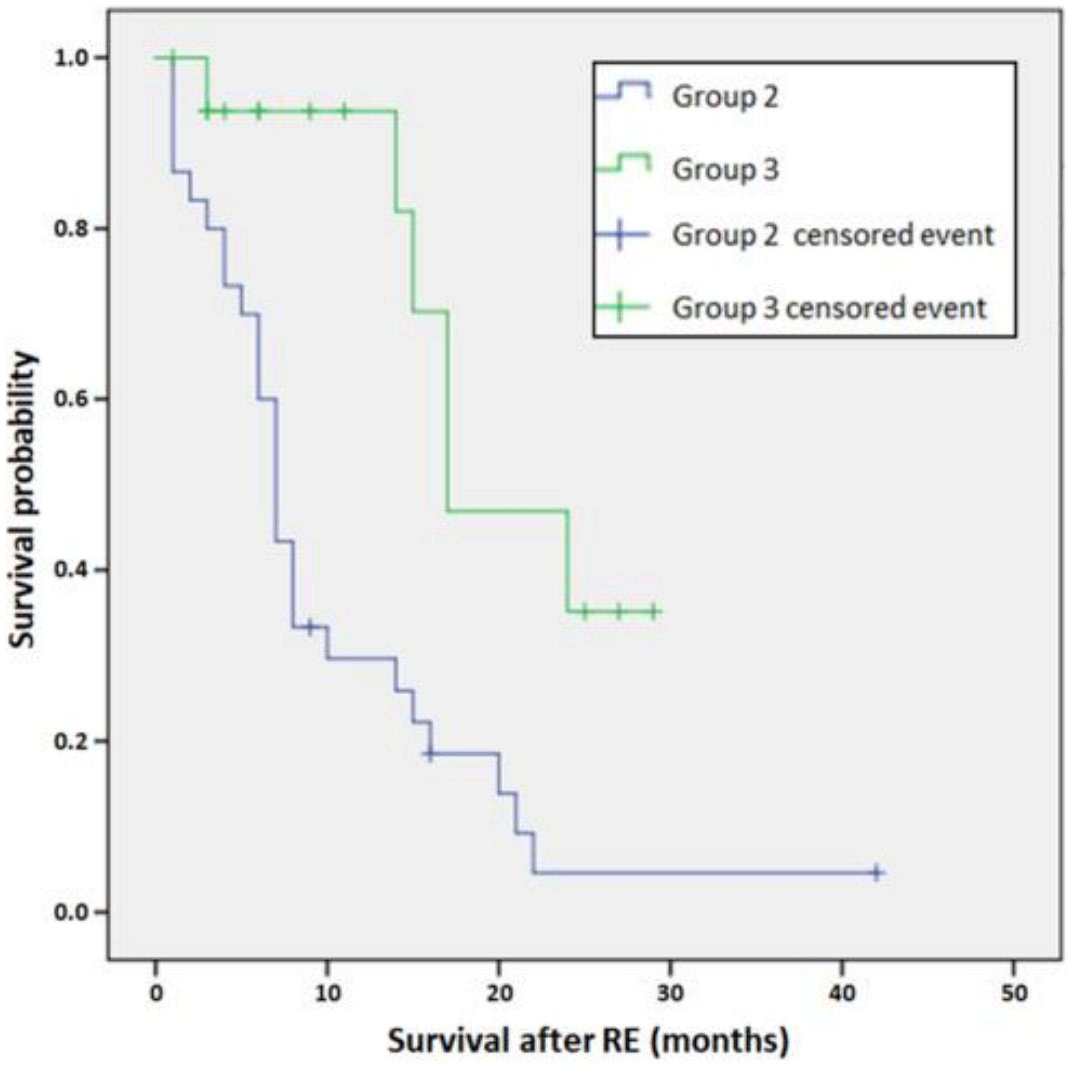

Figure S3. Overall survival depending on time-point of treatment. Kaplan Meier survival analysis calculated from the date of radioembolization. Median survival (95%CI) was 7.0 (5.9–8.1) months in group 2 ($GP2^{SP}$) versus 17.0 (8.6–25.5) months in Group 3 ($GP3^{MP}$). Group 2, standard prophylaxis 23.06.2010 – 14.01.2014 ($n = 30$); Group 3, modified prophylaxis 15.01.2014–28.04.2015 ($n = 17$); RE, radioembolization. See also table 5.

Table S1. Laboratory parameters at baseline and follow-up (Part II).

Variable (Normal Range)		Standard Prophylaxis (n = 48)	Modified Prophylaxis (n = 30)	p-Value (between Group) [a]	p-Value (Baseline vs. Follow-Up) [b]
Albumin (35–52 g/L)	Baseline	42.69 ± 3.45	41.67 ± 2.41	0.080	
	Follow-up	38.83 ± 5.35	39.74 ± 4.26	0.484	<0.001 / 0.011
Alkaline phosphatase (0.5–2 µmol/s.L)	Baseline	2.44 ± 1.39	2.29 ± 2.13	0.072	
	Follow-up	3.34 ± 2.31	3.13 ± 2.30	0.583	0.001 / 0.018
Aspartate transaminase (0.17–0.83 µmol/s.L)	Baseline	0.80 ± 0.33	0.71 ± 0.46	0.046	
	Follow-up	1.28 ± 1.52	1.01 ± 0.80	0.321	0.002 / 0.002
Alanine transaminase (0.17–0.83 µmol/s.L)	Baseline	0.64 ± 0.31	0.66 ± 0.57	0.331	
	Follow-up	0.82 ± 0.51	0.68 ± 0.41	0.430	0.007 / 0.290
Cholinesterase (88–215 µmol/s.L)	Baseline	127.53 ± 38.31	127.20 ± 26.14	0.884	
	Follow-up	113.08 ± 38.00	111.47 ± 37.11	0.971	0.001 / 0.003
Prothrombin time in % (>70%)	Baseline	115.72 ± 8.14	103.7 ± 11.72	<0.001	
	Follow-up	112.36 ± 19.08	101.53 ± 14.33	<0.001	0.278 / 0.657
C-reactive protein (<5 mg/dL)	Baseline	17.32 ± 30.87	6.28 ± 8.51	0.003	
	Follow-up	29.06 ± 47.53	14.30 ± 26.68	0.019	0.049 / 0.003
Gamma glutamyltransferase (0.17–1.19 µmol/s.L)	Baseline	2.71 ± 2.56	2.77 ± 3.75	0.199	
	Follow-up	3.23 ± 4.29	2.83 ± 3.37	0.395	0.215 / 0.233
Glutamate dehydrogenase (<120 nmol/s.L)	Baseline	163.66 ± 157.25	182.57 ± 226.21	0.251	
	Follow-up	148.70 ± 146.17	119.70 ± 126.58	0.461	0.267 / 0.727
Thrombocytes (146–328 Gpt/L)	Baseline	276.26 ± 96.12	257.57 ± 78.30	0.253	
	Follow-up	189.36 ± 61.67	184.53 ± 74.27	0.412	<0.001 / <0.001

Mean values +/− 1 SD; [a] Between group comparison, Mann-Whitney U test; [b] Wilcoxon Test Comparison versus baseline, Wilcoxon test (standard prophylaxis / modified prophylaxis); $p < 0.05$ indicates statistical significance.

.

Table S2. Predictors of RILD [1] (n = 78).

Variable	Univariate Analysis		Multivariate Analysis	
	P	**Odds Ratio (95%CI)**	***P***	**Odds Ratio (95%CI)**
Age ≤ 60	0.177	4.40 (0.51–37.73)		
Estrogen receptor positive	0.559	1.91 (0.22–16.75)		
Progesterone receptor positive	0.238	3.65 (0.42–31.44)		
Hormone receptor positive [2]	0.674	1.60 (0.18–14.13)		
Her2 neu positive [3]	0.812	0.77 (0.09–6.86)		
TNBC [4]	0.891	0.86 (0.10–7.73)		
Grading G3 *	0.462	0.53 (0.10–2.85)		
Time ID breast cancer to ID LMBC ≥ 2 years	0.481	0.58 (0.13–2.66)		
Time ID metastatic liver disease to RE ≥ 1 year	0.416	2.00 (0.38–10.63)		
Tumour load ≥ 5%	0.710	1.38 (0.26–7.36)		
Maximum diameter of liver metastases ≥ 3.9 cm	0.416	1.87 (0.41–8.43)		
Pretherapeutic extrahepatic disease	0.897	1.12 (0.21–6.03)		
Pretherapeutic ascites	0.220	4.86 (0.39–60.57)		
Pretherapeutic chemotherapy ≥ 3 lines	0.831	1.18 (0.26–5.33)		
Prior local therapies of liver metastases	0.423	1.87 (0.40–8.66)		
surgery	0.595	1.86 (0.19–18.23)		
interstitial brachytherapy	0.977	0.97 (0.11–8.82)		
radiofrequency ablation	0.342	3.19 (0.29–34.94)		
Bilirubin ≥ 21 µmol/L	0.119	9.86 (0.55–175.38)		
Cholinesterase ≤ 88 µmol/s.L	0.211	1.02 (0.99–1.04)		
Albumin ≤ 35 g/L	0.119	9.86 (0.55–175.38)		
C-reactive protein ≥ 5 mg/dl	0.197	3.00 (0.57–15.90)		
Gamma glutamyltransferase ≥ 1.19 µmol/s.L	0.076	7.00 (0.82–59.92)		
Glutamate dehydrogenase ≥ 120 µmol/s.L	0.234	2.50 (0.55–11.31)		
Alkaline phosphatase ≥ 2 µmol/s.L	0.078	4.50 (0.85–23.91)		
Aspartate transaminase ≥ 0.83 µmol/s.L	0.008	18.79 (2.17–163.01)	0.011	17.12 (1.93–151.95)
Alanine transaminase ≥ 0.83 µmol/s.L	0.111	3.38 (0.76–15.04)		
Thrombocytes ≤ 146 Gpt/L	0.046	7.44 (1.03–53.62)		
ALBI Grade > 1	0.077	5.50 (0.83–36.48)		
Bilobular RE	0.481	0.58 (0.13–2.66)		
Unilobular RE	0.368	2.03 (0.44–9.41)		
Total radiation dose ≥ 1500 MBq	0.588	0.67 (0.15–2.89)		
Chemotherapy during follow-up	0.434	2.00 (0.35–11.33)		
Modified prophylaxis	0.145	0.20 (0.02–1.73)		

Binary logistic regression was performed. $P < 0.05$ indicate statistical significance. [1] including all cases of RILD (n = 8); [2] estrogen- and/or progesterone receptor positive; [3] at least IHC-Score +3; [4] triple-negative breast cancer: estrogen receptor, progesterone receptor and Her2 neu negative; RILD, Radiation induced liver disease; ALBI, albumin-bilirubin grade; MBq, megabecquerel. * missing values: Grading. 4/78.

Table S3. Changes of portal vein and maximal spleen diameter as hallmarks of portal hypertension post RE.

Variable	SP			MP			
	Pre RE	FU	*p*-Value x	Pre RE	FU	*p*-Value x	*p*-Value #
Portal Vein max. Dia mm (IQR)	9.6 (8.6–10.6)	11.3 (10.1–11.9)	<0.001	10.1 (9.2–10.5)	11.4 (10.6–11.8)	<0.001	
Spleen max. Dia mm (IQR)	93.8 (82.9–103.3)	96.8 (91.1–103.7)	<0.001	91.6 (82.9–102.9)	101.8 (86.9–110.4)	<0.001	
Δ Portal vein mm	1.5 (0.5–2.3)			1.3 (0.6–1.8)			0.523
Δ Spleen mm	4.0 (0.5–8.8)			7.1 (2.1–12.8)			0.119

x Wilcoxon Test; # Mann-Whitney U Test.
